# Functional Promoter -94 ins/del ATTG Polymorphism in *NFKB1* Gene Is Associated with Bladder Cancer Risk in a Chinese Population

**DOI:** 10.1371/journal.pone.0071604

**Published:** 2013-08-20

**Authors:** Pengchao Li, Jinbao Gu, Xiao Yang, Hongzhou Cai, Jun Tao, Xuejian Yang, Qiang Lu, Zengjun Wang, Changjun Yin, Min Gu

**Affiliations:** 1 Department of Urology, The First Affiliated Hospital of Nanjing Medical University, Nanjing, China; Biomedical Research Foundation, Academy of Athens, Greece

## Abstract

**Background:**

A functional -94 insertion/deletion polymorphism (rs28362491) in the promoter of the NFKB1 gene was reported to influence *NFKB1* expression and confer susceptibility to different types of cancer. This study aims to determine whether the polymorphism is associated with risk of bladder cancer.

**Materials and methods:**

TaqMan assay was used to determine genotype among 609 cases and 640 controls in a Chinese population. Logistic regression was used to assess the association between the polymorphism and bladder cancer risk, and quantitative real-time polymerase chain reaction was used to determine *NFKB1* mRNA expression.

**Results:**

Compared with the ins/ins/ins/del genotypes, the del/del genotype was associated with a significantly increased risk of bladder cancer [adjusted odd ratio (OR)  = 1.92, 95% confidence interval (CI)  = 1.42–2.59]. The increased risk was more prominent among subjects over 65 years old (OR  = 2.37, 95% CI  = 1.52–3.70), male subjects (OR  = 1.97, 95% CI = 1.40–2.79) and subjects with self-reported family history of cancer (OR  = 3.59, 95% CI  = 1.19–10.9). Furthermore, the polymorphism was associated with a higher risk of developing non-muscle invasive bladder cancer (OR  = 2.07, 95% CI  = 1.51–2.85), grade 1 bladder cancer (OR  = 2.40, 95% CI  = 1.68–3.43), single tumor bladder cancer (OR  = 2.04, 95% CI  = 1.48–2.82) and smaller tumor size bladder cancer (OR  = 2.10, 95% CI  = 1.51–2.92). The expression of *NFKB1* mRNA in bladder cancer tissues with homozygous insertion genotype was higher than that with deletion allele.

**Conclusions:**

In conclusion, the -94 ins/del ATTG polymorphism in *NFKB1* promoter may contribute to the etiology of bladder cancer in the Chinese population.

## Introduction

Bladder cancer, primarily urothelial cell carcinoma (UCC), is the second most common genitourinary malignancy that leads to significant morbidity and mortality [1]. UCC is a heterogeneous disease, with 70% of patients presenting non-muscle invasive tumors and 30% presenting muscle-invasive disease associated with a poor prognosis from distant metastases [2]–[4]. These tumors have a lifelong risk of recurrence but are generally not life threatening. Risk factors for bladder tumorigenesis can be classified into three subsets: genetic and molecular abnormalities, chemical or environmental exposures, and chronic irritation [5], [6]. Although many people are exposed to the above-mentioned risk factors, only a fraction of exposed individuals develops UCC. Thus, individual variations in the susceptibility to risk factors leading to bladder carcinogenesis may exist.

Nuclear factor-κB (NF-κB) was first identified in 1986 as a B-cell factor that binds to a site in the enhancer region of the gene encoding the immunoglobulin κ light chain [7]. Since then, NF-κB has been shown to regulate the transcription of many genes for immune response, cell adhesion, differentiation, proliferation, angiogenesis, cellular stress reactions, tumorigenesis, and cell survival and apoptosis [4]. Several investigators have reported the constitutive activation of NF-κB in various tumor cells and cell lines, such as breast cancer [8], colorectal cancer [9], [10], lung cancer [11], [12], and pancreatic cancer [13]. The activation of NF-κB was reported to be potentially associated with bladder cancer growth by protecting the cancer cells from apoptotic cell death [14]. NF-κB is a heterodimer of the Rel family with five members, namely, RelA, RelB, c-Rel, p50 (NF-κB1), and p52 (NF-κB2); it is also the point of convergence of some metabolic and oncogenic pathways [15].

The *NFKB1* gene, which is located at human chromosome 4q24, encodes protein p50 that could act as a transcription factor to regulate its target gene transcription [16], [17]. The -94 ins/del ATTG polymorphism (rs28362491) in *NFKB1* promoter reportedly elicits a regulatory effect on the *NFKB1* gene and plays a role in the susceptibility of individuals to various malignancies, including gastric cancer [18], ovarian cancer [19], prostate cancer [20], and oral squamous cell carcinoma [21]. Riemann *et al.* [22] reported that *NFKB1* (p50) mRNA expression is higher in tumor tissues with the ins/ins genotype than in those with the ins/del + del/del genotype. They also found that patients with homozygous deletion possess a statistically higher risk of tumor recurrence than carriers with one or more insertion alleles in non-muscle invasive bladder cancer. However, in their study, no association was found between the -94 ins/del ATTG polymorphism in *NFKB1* promoter and bladder cancer risk [22].

In the present study, we hypothesized that the -94 ins/del ATTG polymorphism in *NFKB1* promoter is associated with bladder cancer risk. This hypothesis was tested in our ongoing, hospital-based, case-control study in a Chinese population.

## Materials and Methods

### Ethics Statement

The study was approved by the Institutional Review Board of the First Affiliated Hospital of Nanjing Medical University, Nanjing, China. Written informed consent was obtained from all participants involved in this study.

### Patients and controls

The participants of this study included 609 bladder cancer patients and 640 age-matched controls. All subjects were from the Han population living in Jiangsu province and Anhui province of Eastern China. Bladder cancer patients were recruited from July 2006 to July 2012 at the Department of Urology, the First Affiliated Hospital of Nanjing Medical University. Patients with histories of cancer, metastasized cancer from other or unknown origins, and previous radiotherapy or chemotherapy were excluded. The diagnosis of bladder cancer was confirmed by histopathologic analysis. Cancer-free control individuals were frequency matched for age (±5 years) and sex, and were genetically unrelated to the cases. All controls were recruited from healthy subjects who were seeking physical examination in the outpatient department of the same hospital. Controls were excluded if they had symptoms suggestive of bladder cancer, such as hematuria. Prior to recruitment, all subjects were interviewed in person to collect demographic data and clinical characteristics, including age, gender, race, tobacco smoking, alcohol drinking, and self-reported family history of cancer. According to tumor-node-metastasis classification for the stage (2002 International Union Against Cancer), the clinical stage at the time of diagnosis was classified into two subgroups: non-muscle invasive group (pTa-pT1) and muscle invasive group (pT2-pT4). According to histopathologic grade (WHO 1973, grading of urothelial papilloma), the patients were classified into three subgroups: grades 1, 2, and 3. Individuals who smoked daily for >1 year were defined as ever smokers, and the rest were considered as never smokers. Individuals who drank at least three times per week for more than 6 months were defined as ever drinkers, and the rest were considered as never drinkers.

### DNA extraction and polymorphism genotyping

Genomic DNA of each individual was extracted from 150 μl EDTA-anticoagulated peripheral blood samples using a DNA extraction kit (Tiangen Biotech, Beijing, China) following the manufacturer's instructions. The -94 ins/delATTG polymorphism in *NFKB1* promoter was genotyped using TaqMan single nucleotide polymorphism (SNP) Genotyping Assays (Applied Biosystems, Foster City, CA, USA), and SDS 2.4 software was used for allelic discrimination. The primers, probes, and reaction conditions for each SNP are available upon request. Amplification was performed under the following conditions: 50°C for 2 min; 95°C for 10 min; and 45 cycles of 95°C for 15 s and 60°C for 1 min. For quality control, four negative controls were included in each plate, and 5% of the samples were randomly selected for repeated genotyping. The results were 100% concordant.

### Quantitative real-time PCR

RNA from 35 frozen bladder cancer tissues (n = 8 for ins/ins genotype and n = 27 for carriers of del allele) was extracted using standard methods (RNeasy kit, Qiagen). A sample of 1 μg total RNA was used for cDNA synthesis with oligo-dT primers (Invitrogen, Karlsrule, Germany) and superscript II reverse transcriptase (Takara Bio, Shiga, Japan). PCR was performed using PCR Master (Roche, Mannheim, Germany) with the following primers of *NFKB1*: 5′-GTGAAGGCCCATCCCATGGT-3′ (forward) and 5′-TGTGACCAACTGAACAATAACC-3′ (reverse) and the primers for *β-actin* were 5′-ACTGGAACGGTGAAGGTGAC-3′ (forward) and 5′-AGAGAAGTGGGGTGGCTTTT-3′ (reverse). PCR in duplicates was done on a 384-well ABI 7900HT Real-Time PCR System (Applied Biosystems, Foster City, CA, USA). The relative expression of *NFKB1* in relation to *β-actin* was calculated using the formula *NFKB1*/*β-actin*  = 2^−ΔCt^.

### Statistical analysis

The frequency distributions of the selected demographic variables and each allele and genotype of the -94 ins/del ATTG polymorphism in *NFKB1* promoter between the cases and controls were evaluated using χ^2^-type distribution. Hardy-Weinberg equilibrium (HWE) was tested using a goodness-of-fit χ^2^ test. Unconditional univariate and multivariate logistic regression analyses were conducted to calculate the crude and adjusted odds ratios (ORs) and their 95% confidence intervals (CIs) for bladder cancer risk. Interaction was tested using a multiplicative interaction term included in the multivariate model. To explore potential interactions between the polymorphism and tobacco smoking, we assessed a multiplicative gene – environment interaction by logistic regression analysis, including the main effect variables and their product terms. All analyses were carried out using SPSS 13.0 statistical software. A two-side *P* value of less than 0.05 was considered to indicate statistical significance.

## Results

### Characteristics of the study population

The frequency distributions of the selected characteristics of the cases and controls are shown in Table 1. The cases and controls was showed to be adequately matched on age and sex (*P* = 0.180 for age and *P* = 0.606 for sex). More ever smokers were found among the cases than the controls (47.9% vs. 38.0%; *P*<0.001). No significant difference in drinking status was found between the cases and controls (*P* = 0.169). In addition, the frequency of relatives with cancer was higher in the cases than in the controls (27.8% vs. 6.6%; *P*<0.001). These variables were further adjusted in the multivariate logistic regression analysis to assess the main effect of the -94 ins/del ATTG polymorphism in *NFKB1* promoter on bladder cancer risk. The clinicopathological characteristics of 609 bladder cancer cases was listed in Table 1.

**Table 1:** Frequency distribution of selected variables of the bladder cancer cases and controls.

Variables	Cases (n = 609)		Controls (n = 640)		*P* a
	n	%	n	%	
**Age (year)**					
<65	289	47.5	328	51.2	
≥65	320	52.5	312	48.8	0.180
**Sex**					
Male	484	79.5	501	78.3	
Female	125	20.5	139	21.7	0.606
**Smoking status**					
Never	317	52.1	397	62.0	
Ever	292	47.9	243	38.0	0.001
**Drinking status**					
No	418	68.6	462	72.2	
Yes	191	31.4	178	27.8	0.169
**Family history of cancer**					
No	440	72.2	598	93.4	
Yes	169	27.8	42	6.6	<0.001
**Tumor stage**					
Non-muscle invasive	466	76.5			
Muscle invasive	143	23.5			
**Tumor grade**					
Grade 1	298	48.9			
Grade 2	194	31.9			
Grade 3	117	19.2			

a Student's t-test for age distribution between the cases and controls; two-sided χ^2^ test for other selected variables between the cases and controls.

### Association between the -94 ins/del ATTG polymorphism in *NFKB1* promoter and bladder cancer risk

The genotype and allele frequency distributions of the -94 ins/del ATTG polymorphism in *NFKB1* promoter among the cases and controls, as well as their associations with bladder cancer risk, are presented in Table 2. The genotype frequencies in the controls conformed to HWE (*P* = 0.164). The frequencies of the ins/ins, ins/del, and del/del genotypes were 31.0%, 44.2%, and 24.8% among the cases, respectively, and 34.8%, 50.6%, and 14.5% among the controls, respectively (*P*<0.001). Using the ins/ins and ins/del genotypes as reference, we found that the del/del genotype was associated with a statistically significant increased risk of bladder cancer (*P*<0.001, OR  = 1.92, 95% CI  = 1.40–2.59). A similar result was observed in the del/del genotype compared with the ins/ins genotype (*P*<0.001, OR  = 2.10, 95% CI  = 1.48–2.97).

**Table 2:** Genotype and allele frequencies of the *NFKB1* promoter -94 ins/del ATTG polymorphism among the bladder cancer cases and controls.

Genotypes	Cases (n = 609)		Controls(n = 640)		*P* a	Crude OR (95%CI)	*P* b	Adjusted OR (95%CI) b
	n	%	n	%				
**ins/ins**	189	31.0	223	34.8		1.00 (reference)		1.00 (reference)
**ins/del**	269	44.2	324	50.6	0.873	0.98 (0.76–1.27)	0.672	1.06 (0.81–1.38)
**del/del**	151	24.8	93	14.5	<0.001	1.92 (1.37–2.68)	<0.001	2.10 (1.48–2.97)
**ins/ins**	189	31.0	223	34.8		1.00 (reference)		1.00 (reference)
**ins/del + del/del**	420	69.0	417	65.2	0.152	1.19 (0.93–1.52)	0.058	1.27 (0.99–1.64)
**ins/ins + ins/del**	458	75.2	547	85.5		1.00 (reference)		1.00 (reference)
**del/del**	151	24.8	93	14.5	<0.001	1.94 (1.44–2.61)	<0.001	1.92 (1.42–2.59)
**ins allele**	647	53.1	770	60.2				
**del allele**	571	46.9	510	39.8	<0.001			

Abbreviations: CI, confidence interval; OR, odds ratio.

a Two-sided χ^2^ test for either genotype distributions or allele frequencies between the cases and controls.

b Adjusted for age, gender, smoking, drinking status and family history of cancer in logistic regression model.

### Stratified analysis of the -94 ins/del ATTG polymorphism in *NFKB1* promoter and bladder cancer risk

We further assessed the effect the -94 ins/del ATTG polymorphism in *NFKB1* promoter on bladder cancer risk stratified by age, sex, smoking status, drinking status, and self-reported family history of cancer. As shown in Table 3, the increased risk was more pronounced among older subjects (*P*<0.001, OR  = 2.37, 95% CI  = 1.52–3.70), male subjects (*P*<0.001, OR  = 1.97, 95% CI  = 1.40–2.79) and subjects with self-reported family history of cancer (*P* = 0.024, OR = 3.59, 95% CI  = 1.19–10.9) (Table 3).

**Table 3:** Stratification analyses between *NFKB1* promoter –94 ins/del ATTG polymorphism and risk of bladder cancer.

Variables		Genotypes (cases/controls)							
	Cases/Controls	ins/ins + ins/del		del/del		*P* a	Crude OR (95%CI)	*P* b	Adjusted OR (95%CI) b
		n	%	n	%				
**Total**	609/640	458/547	75.2/85.5	151/93	24.8/14.5	<0.001	1.94 (1.44–2.61)	<0.001	1.92 (1.42–2.59)
**Age(year)**									
<65	289/328	217/273	75.1/83.2	72/55	24.9/16.8	0.013	1.65 (1.09–2.49)	0.038	1.55 (1.02–2.35)
≥65	320/312	241/274	75.3/87.8	79/38	24.7/12.2	<0.001	2.36 (1.52–3.71)	<0.001	2.37 (1.52–3.70)
**Sex**									
Male	484/501	370/433	76.4/86.4	114/68	23.6/13.6	<0.001	1.96 (1.39–2.77)	<0.001	1.97 (1.40–2.79)
Female	125/139	88/114	70.4/82.0	37/25	29.6/18.0	0.026	1.92 (1.03–3.58)	0.092	1.73 (0.92–3.27)
**Smoking status**									
Never	317/397	227/338	71.6/85.1	90/59	28.4/14.9	<0.001	2.27 (1.55–3.35)	0.004	1.79 (1.20–2.67)
Ever	292/243	231/209	79.1/86.0	61/34	20.9/14.0	0.038	1.62 (1.00–2.65)	0.013	1.84 (1.14–2.98)
**Drinking status**									
No	418/462	323/400	74.9/86.6	95/62	25.1/13.4	<0.001	1.90 (1.32–2.75)	0.003	1.77 (1.21–2.58)
Yes	191/178	135/147	70.7/82.6	56/31	29.3/17.4	0.007	1.97 (1.17–2.35)	0.020	1.87 (1.10–3.17)
**Family history of cancer**									
No	440/598	336/509	76.4/85.1	104/89	23.6/14.9	<0.001	1.77 (1.28–2.46)	<0.001	1.79 (1.30–2.46)
Yes	169/42	122/38	77.2/90.5	47/4	27.8/9.5	0.013	3.66 (1.21–14.8)	0.024	3.59 (1.19–10.9)

Abbreviations: CI, confidence interval; OR, odds ratio.

a Two-sided χ^2^ test for either genotype distributions or allele frequencies between the cases and controls.

b Adjusted for age, gender, smoking, drinking status and family history of cancer in logistic regression model.

### Association between the -94 ins/del ATTG polymorphism in *NFKB1* promoter and clinicopathological characteristics of bladder cancer patients

When compared with control group (Table 4), the risk of bladder cancer significantly increased in the del/del genotype compared with the ins/del + ins/ins genotype in non-muscle invasive cases (*P*<0.001, OR  = 2.07, 95% CI  = 1.51–2.85), grade 1 bladder cancer cases (*P*<0.001, OR  = 2.40, 95% CI  = 1.68–3.43), single tumor cases (*P*<0.001, OR  = 2.04, 95% CI  = 1.48–2.82) and cases with smaller tumor size (*P*<0.001, OR  = 2.10, 95% CI  = 1.51–2.92). No significant association was observed in the stratification of tumor grade and stage of bladder cancer cases. (Table 5).

**Table 4:** The associations between *NFKB1* promoter –94 ins/del ATTG polymorphism and the development of bladder cancer.

Variables	Genotypes				*P* a	Crude OR (95%CI)	*P* b	Adjusted OR (95%CI) b
	ins/ins + ins/del		del/del					
	n	%	n	%				
**Controls(n = 640)**	547	85.5	93	14.5		1.00 (Reference)		1.00 (Reference)
**Cases(n = 609)**								
**Tumor Stage**								
Non-muscle invasive	347	74.5	119	25.5	<0.001	2.02 (1.47–2.76)	<0.001	2.07 (1.51–2.85)
muscle invasive	111	77.6	32	22.4	0.021	1.70 (1.04–2.70)	0.029	1.71 (1.06–2.76)
**Tumor Grade**								
Grade 1	221	74.2	77	25.8	<0.001	2.05 (1.44–2.92)	<0.001	2.40 (1.68–3.43)
Grade 2	149	76.8	45	23.2	0.004	1.78 (1.16–2.69)	0.013	1.75 (1.12–2.72)
Grade 3	88	75.2	29	24.8	0.006	1.94 (1.16–3.17)	0.038	1.74 (1.03–2.94)
**Number**								
Single	326	74.8	110	25.2	<0.001	1.98 (1.44–2.73)	<0.001	2.04 (1.48–2.82)
Multiple	132	76.3	41	23.7	0.004	1.83 (1.17–2.80)	0.009	1.81 (1.16–2.83)
**Size**								
<3cm	287	73.4	104	26.6	<0.001	2.13 (1.54–2.95)	<0.001	2.10 (1.51–2.92)
≥3cm	171	78.4	47	21.6	0.015	1.62 (1.07–2.42)	0.011	1.71 (1.13–2.59)

Abbreviations: CI, confidence interval; OR, odds ratio.

a Two-sided χ^2^ test for either genotype distributions or allele frequencies between the cases and controls.

b Adjusted for age, gender, smoking, drinking status and family history of cancer in logistic regression model.

**Table 5:** *NFKB1* promoter –94 ins/del ATTG polymorphism and clinicopathological characteristics in patients with bladder cancer.

Clinicopathological Characteristics		Genotypes n(%)		*P* a	Adjusted OR (95%CI) a
		ins/ins + ins/del	del/del		
**Tumor Stage**	**Non-muscle invasive**	347 (74.5)	119 (25.5)		1.00 (reference)
	**Muscle invasive**	111 (77.6)	32 (22.4)	0.645	0.90 (0.57–1.41)
**Tumor Grade**	**Grade 1**	221 (48.3)	77 (51.0)		1.00 (reference)
	**Grade 2**	149 (32.5)	45 (29.8)	0.865	1.04 (0.66–1.63)
	**Grade 3**	88 (19.2)	29 (19.2)	0.829	1.06 (0.63–1.79)
**Number**	**Single**	326 (74.7)	110 (25.2)		1.00 (reference)
	**Multiple**	132 (76.3)	41 (23.7)	0.440	0.85 (0.56–1.29)
**Size**	**≤3cm**	287 (73.4)	104 (26.6)		1.00 (reference)
	**<3cm**	171 (78.4)	47 (21.6)	0.220	0.78 (0.52–1.16)

Abbreviations: CI, confidence interval; OR, odds ratio.

a Adjusted for age, gender, smoking, drinking status and family history of cancer in logistic regression model.

### Interaction between the -94 ins/del ATTG polymorphism in *NFKB1* promoter and tobacco smoking

Compared with non-smokers with the ins/ins and ins/del genotypes, smokers with the del/del genotype had significantly increased risk of bladder cancer (*P*<0.001, OR  = 2.53, 95% CI  = 1.55–4.11) (Table 6). Given that the del/del genotype is associated with an increased risk in smokers, we evaluated whether smoking status is associated with the -94 ins/del ATTG polymorphism in *NFKB1* promoter. We did not observe a multiplicative interaction effect between the polymorphism and smoking status (*P* = 0.783) (Table 6).

**Table 6:** Interaction analyses of the *NFKB1* promoter –94 ins/del ATTG polymorphism and tobacco smoking.

Smoking status	Genotypes	Cases		Controls		*P* a	Crude OR (95%CI)^a^	*P* b	Adjusted OR (95%CI) b
		n	%	n	%				
Nonsmokers	ins/ins + ins/del	227	37.3	338	52.8		1.00 (reference)		1.00 (reference)
Nonsmokers	del/del	90	14.8	59	9.2	0.001	2.27 (1.55–3.35)	0.004	1.79 (1.20–2.67)
Smokers	ins/ins + ins/del	231	37.9	209	32.7	<0.001	1.65 (1.27–2.13)	0.002	1.56 (1.17–2.07)
Smokers	del/del	61	10.0	34	5.3	<0.001	2.67 (1.66–4.33)	<0.001	2.53 (1.55–4.11)
***P*** **_Interaction_ (multiplicative)**									0.783

Abbreviations: CI, confidence interval; OR, odds ratio.

a Two-sided χ^2^ test for either genotype distributions or allele frequencies between the cases and controls.

b Adjusted for age, gender, smoking, drinking status and family history of cancer in logistic regression model.

### Association between the -94 ins/del ATTG polymorphism in *NFKB1* promoter and *NFKB1* mRNA expression

**Figure 1:**
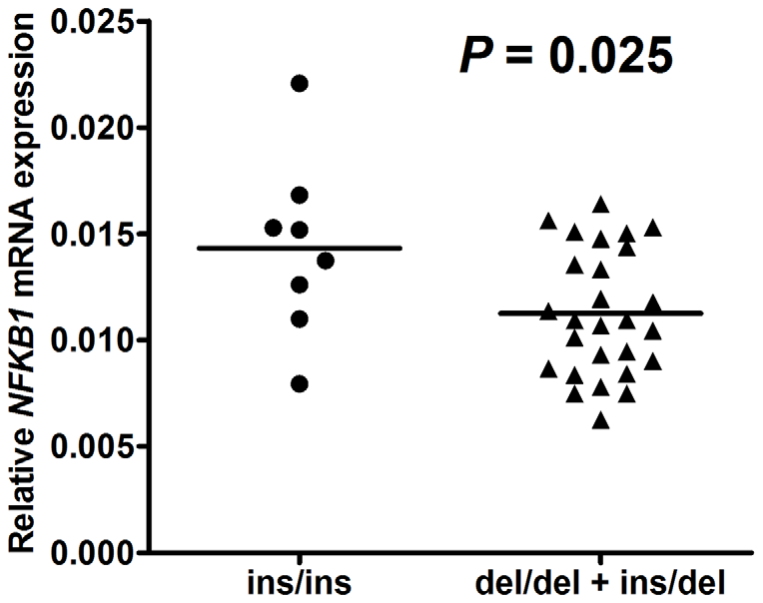
Analysis of *NFKB1* mRNA expression in different genotypes of bladder cancer tissue with mean values (horizontal lines, mean values).

The amount of relative *NFKB1* mRNA in bladder cancer tissues obtained from the homozygous insertion genotype (n = 8) was higher than that in tissues from the ins/del + del/del genotype (n = 27) (*P* = 0.025), even though there was significant overlap between the two groups (Figure 1).

## Discussion

In this study, we identified an association between the -94 ins/del ATTG polymorphism in *NFKB1* promoter and bladder cancer risk in a Chinese population. We observed that individuals with the del/del genotype have 1.92 times greater risk for bladder cancer than those with the ins/ins and ins/del genotypes (*P*<0.001). Furthermore, individuals homozygous for del have higher bladder cancer risk than those homozygous for ins (OR  = 2.10, *P*<0.001). Besides, individuals with the del/del genotype was also associated with an increased risk of developing non-muscle invasive, grade 1, single tumor and smaller tumor size bladder cancer.

As a subunit of the NF-κB complex, p50 is encoded by the *NFKB1* gene [16]. The over-expression of p50 (NF-κB1) was observed in various malignancies [22], [23], including non-small cell lung carcinoma, colon cancer, prostate cancer, breast cancer, bone cancer, and brain cancer. Considering that p50 over-expression is frequently observed in various tumor tissues, p50 is potentially involved in tumorigenesis.

Genetic variation plays a critical role in determining cancer risk, and several studies have investigated the association between the -94 ins/del ATTG polymorphism in *NFKB1* promoter and cancer risk [18]–[21]. To date, only two studies have attempted to evaluate the association between this polymorphism and bladder cancer risk [24], [25]. Riemann *et al*. [24] reported that the polymorphism is not associated with an increased susceptibility to transitional cell carcinoma of the bladder in Caucasians. In our study, the del/del genotype was found to be associated with increased risk of bladder transitional cell carcinoma, including the non-muscle invasive and muscle invasive types. The disparity of results could be attributed to the different ethnicity of the study population. Tang *et al.* [25] studied a southwest Chinese population of 207 cases and reported that individuals with the ins/ins genotype have increased risk of non-muscle invasive transitional cell carcinoma of the bladder. Although the research population of Tang *et al.* [25] and present study were both Chinese Han population, the geographical distribution between our study population and theirs was different, which could result in divergent genetic backgrounds. Therefore, it might be a probable explaination for the contradictory results between our study and theirs. Moreover, the sample size in our study was moderately larger than two aforementioned bladder cancer studies, which might also partly explain the discrepancy.

The probable mechanism behind the observed association may be linked to the expression and activity of p50 (NF-κB1), which regulates important cellular events such as apoptosis and cell death independent of the NF-κB complex [17]. Using a reporter assay, a previous study found that the –94 ins/del ATTG polymorphism has regulatory influence on *NFKB1* gene expression and that the activity of the ins allele is twice as high as that of the del allele [26]. Riemann *et al.* [24] reported that p50 (*NFKB1*) expression in bladder cancer tissues is higher in individuals with the ins/ins genotype than in those carrying the del allele. In the current study, we also identified a positive association between the ins/ins genotype and increased p50 (*NFKB1*) expression in bladder cancer tissues. The difference in the expression of p50 (*NFKB1*) in bladder cancer tissues between the ins/ins genotype and carriers of del allele may indicate the regulatory effect of the -94 ins/del ATTG polymorphism in *NFKB1* promoter on p50 (*NFKB1*) expression. Previous works revealed that p50 induces MEKK4 activation by proteasome-dependent GADD45α degradation [27], [28]. Furthermore, the activation of MEKK4 specifically activates the JNK signaling pathway, which is involved in environmental stress-induced cell apoptosis [29], [30]. Therefore, the provocative explanation for the observed association between the -94 ins/del ATTG polymorphism and bladder cancer risk is that the del/del genotype induces the development of bladder cancer by down-regulating the expression of p50 (*NFKB1*), which plays a role in inducing cell apoptosis through the aforementioned mechanism.

Homodimers of p50 (NF-κB1) can repress the transcription of genes regulated by NF-κB [31]. The increased formation of the p50-p50 complex arising in part from enhanced p50 (*NFKB1*) expression [32], [33] suggests a reduced ability to promote *TNF-α* expression in cells [34]–[36]. The expression levels of many genes regulated by NF-κB, such as *TNF-α*, were also proposed to be higher in *NFKB1* (p50) knockout mice [37], [38]. The cytokine TNF-α promotes tumor proliferation by inducing the release of matrix metalloproteinase (MMP) [39]–[43], particularly the gelatinases MMP-2 and MMP-9, which promote tumor growth and metastasis [44], [45]. Thus, we speculate that the ins/ins genotype can reduce the transcription of genes regulated by NF-κB, such as *TNF-α*, by enhancing p50 expression. Conversely, the del/del genotype can promote the proliferation of bladder cancer through the transcriptional activation of *TNF-α* caused by the inhibitory effect of the del/del genotype on the formation of p50 homodimers [44], [45].

Subgroup analysis according to clinicopathological characteristics, including stage, grade, tumor number, and tumor size, might help in identifying prognostic factors involved in various bladder cancer progression pathways [46]. Riemann *et al.* [24] reported that the time-to-recurrence is significantly shorter in patients with the del/del genotype than in those carrying the ins allele. A large majority (70%) of bladder cancer was considered to be of the non-muscle invasive type, which tends to recur and seldom progresses [2]. In this study, the del/del genotype was also related to an increased risk of developing non-muscle invasive or grade 1 bladder cancer (Table 4). This finding is in accordance with the study of Riemann *et al.* [24]. However, further follow-up studies with larger sample sizes are needed to validate these results. Furthermore, the del/del genotype was associated with a more pronounced risk of bladder cancer in the elder subject and subject with self-reported family history of cancer compared with the ins/ins + ins/del genotype (Table 3). Age is now widely accepted as the greatest single risk factor in developing bladder cancer [47], [48]. Several theories have been proposed to explain the relationship between carcinogenesis and ageing [49]. As individuals continue to age, they experience cumulative environmental exposure to carcinogens, as well as the accumulation of cellular events, such as genomic instability. The patients with self-reported family history of cancer was also believed to suffer from an increased risk of bladder cancer [50]. This fact explains the stronger association between the del/del genotype and bladder tumorigenesis in the elder subgroup and subgroup with self-reported family history of cancer. In the present study, the risk was found to be more significant in males. Male patients were considered to be more exposed to tobacco smoking and other risk factors implicated in the etiology of bladder cancer, which may explain why men are more susceptible to bladder cancer.

Several potential limitations of the present study should be considered. First, our study was a hospital-based case-control study. Thus, we cannot exclude the chance of selection bias to subjects that may have been associated with a particular genotype. Second, the sample size (609 cases and 640 controls) in this study may not be large enough to detect small effects from very low-penetrance SNPs and evaluate gene – environment interaction adequately. However, under the current sample size, we have 99.5% power to detect an OR of ≥1.92 with an exposure frequency of 39.8% at the 0.05 significance level.

In conclusion, we identified an association between the -94 insertion/deletion ATTG polymorphism in *NFKB1* promoter and bladder cancer risk in a Chinese population. The del/del genotype was found to be associated with an increased risk of bladder cancer.
